# Long-term exposure to polystyrene microplastics triggers premature testicular aging

**DOI:** 10.1186/s12989-023-00546-6

**Published:** 2023-08-28

**Authors:** Deyi Wu, Meng Zhang, Ting Ting Bao, Hainan Lan

**Affiliations:** 1 College of Animal Science and Technology, Jilin Agricultural University, Changchun, 130118 China; 2 The Second Affiliated Hospital of Guangxi Medical University, Nanning, 530005 China

**Keywords:** Polystyrene microplastics, Premature testicular aging, TM4 cells, ROS, NF-κB

## Abstract

**Background:**

Plastic pollution is greatly serious in the ocean and soil. Microplastics (MPs) degraded from plastic has threatened animals and humans health. The accumulation of MPs in the tissues and blood in animals and humans has been found. There is therefore a need to assess the toxicological effects of MPs on the reproductive system.

**Results:**

In this study, we explored the effect of polystyrene microplastics (PS-MPs) on premature testicular aging in vitro and in vivo. In vitro, we found that testicular sertoli cells (TM4 cells) was prematurely senescent following PS-MPs treatment by the evaluation of a range of aging marker molecules (such as Sa-β-gal, p16 and 21). TM4 cells were then employed for in vitro model to study the potential molecular mechanism by which PS-MPs induce the premature senescence of TM4 cells. NF-κB is identified as a key molecule for PS-MPs-induced TM4 cellular senescence. Furthermore, through eliminating reactive oxygen species (ROS), the activation of nuclear factor kappa B (NF-κB) was blocked in PS-MPs-induced senescent TM4 cells, indicating that ROS triggers NF-κB activation. Next, we analyzed the causes of mitochondrial ROS (mtROS) accumulation induced by PS-MPs, and results showed that Ca^2+^ overload induced the accumulation of mtROS. Further, PS-MPs exposure inhibits mitophagy, leading to the continuous accumulation of senescent cells. In vivo, 8-week-old C57 mice were used as models to assess the effect of PS-MPs on premature testicular aging. The results illustrated that PS-MPs exposure causes premature aging of testicular tissue by testing aging markers. Additionally, PS-MPs led to oxidative stress and inflammatory response in the testicular tissue.

**Conclusion:**

In short, our experimental results revealed that PS-MPs-caused testicular premature aging is dependent on Ca^2+^/ROS/NF-κB signaling axis. The current study lays the foundation for further exploration of the effects of microplastics on testicular toxicology.

**Supplementary Information:**

The online version contains supplementary material available at 10.1186/s12989-023-00546-6.

## Background

Plastic and plastic-based products are widely utilized for packaging and transport worldwide because of their convenience and utility. According to statistics, around 8 million tons plastic waste enters the ocean each year, and global plastic production is expected to reach around 33 billion tons by 2050 [1]. Plastic and plastic-based products are manufactured in various material types, including polystyrene monomer, polyethylene terephthalate, polypropylene and polyvinyl chloride. Microplastics (MPs) are small particles, ranging from 0.1 μm to 5 mm in diameter, that are degraded from plastic through prolonged UV exposure or biodegradation [2]. MPs have been detected in the air, ocean and soil due to the indiscriminate disposal of plastic. The imbalance between the rate of generation and the rate of biodegradation of MP has already caused a serious burden on the environment. The main routes of exposure to MP in human are through inhalation, oral intake, dermal contact and trophic transfer in the food chain [3]. It is estimated that MP ingestion were 15–287 g/ person each year base on mass or weight according to Senathirajah et al. [4].

The severity of global MPs pollution has caught the attention of scientists. Numerous studies have revealed the toxicity of PS-MPs. The accumulation of PS-MPs with particle size with 5 μm was observed in gills, liver and gut of zebrafish, which led to inhibit lipolysis and energy metabolism [5]. In addition, the accumulated PS-MPs (5 and 50 μm diameter) elevated levels of inflammation and changed the relative abundances of microbiome in larval zebrafish [6, 7]. Apart from fish, PS-MP can also be uptaken by shellfish and *Daphnia* and cause damage to the organism [8, 9]. Studies on the toxicity of PS-MPs on mammals have been carried out. The latest study has revealed that MPs (≥ 0.7 μm) can be absorbed by digestive tract and accumulated in human blood [10]. Qiao et al. and Liang et al. found that the ingested PS-MPs can result in intestinal barrier dysfunction and intestinal flora dysbiosis in mice [11, 12]. Additionally, PS-MPs administration triggers liver fibrosis in mice through activating cGAS/STING signaling pathway [13]. In vitro, MPs exposure has also been reported to exert detrimental effects. For example, PS-MP exposure causes mitochondrial and lysosomal damage in rat basophilic leukemia cells [14]. High dose of MPs accumulation can lead to cell apoptosis and death [15].

The reproductive system is greatly sensitive to environmental pollution. The effects of MPs on the reproductive system in aquatic organisms and mammals have been reported. PS-MPs exposure with particle size of 5 μm for 4 weeks reduced testosterone levels, hatching and survival rates of offspring in freshwater prawn [16]. Jin et al. revealed a significant decrease in sperm quality after PS-MPs exposure [17]. Additionally, PS-MPs exposure led to the disruption of blood-testis barrier via inducing imbalance of mTORC1 and mTORC2 which was regulated by ROS [18]. Also, PS-MPs exposure induced the inflammation of ovaries and reduced the quality of oocytes in female mice [19]. Xie et al. found that the toxicity of PS-MPs to reproductive system is mediated by p38 signaling pathway [20]. However, the effect of PS-MPs on premature testicular aging and the molecular mechanism remains unclear.

PS is one of the most frequently used plastic in daily life. PS-MPs polymerized from styrene monomers is usually used to make disposable products, such as disposable lunch box and food packaging due to their thermoplasticity [21]. For following study, the effects of PS-MPs (1 μm) on the toxicology of testicular tissues were explored. The aim of the study was to investigate the effect of PS-MPs on premature testicular aging and the molecular mechanism. The results showed that PS-MPs-caused premature testicular aging was modulated by oxidative stress-mediated NF-κB signaling pathway. Elevated ROS level was attributed to increased Ca^2+^ overload in the mitochondrial. Our study is the first to evaluate the effect of PS-MPs on premature aging of testicular tissue, laying the groundwork for subsequent research on the toxicity of PM-MPs on reproductive system.

## Methods

### Reagents and antibodies

PS-MPs (1 μm) and fluorescent-labelled PS-MPs (1 μm) were purchasedfrom Tianjin Bestra Chromatography Technology Development Center (25 mg/mL stock solution). The stock solution was diluted to the specified concentration using complete medium. The monoclonal antibodies, p16 (ab51243; 1:50 dilution for IHC, 1:1000 dilution for WB), Ki67 (ab15580; 1:200 dilution), γ-H2AX (ab81299; 1:200 dilution), 53BP1 (ab175933; 1:200 dilution), IL-16 (ab180792; 1:100 dilution), 4-Hydroxynonenal (ab48506; 1:100 dilution), H3K9me3 (ab8898; 1:1000 dilution), Histone (ab1791; 1:1000 dilution), IL-6 (ab9324; 1:1000 dilution), IL-8 (ab18672; 1:1000 dilution), TNF-α (ab1793; 1:1000 dilution), were obtained from Abcam (US). The monoclonal antibodies, α-SMA (19,245; 1:500 dilution), p53 (48,818; 1:100 dilution for IHC, 1:1000 dilution for WB), p21 (2947; 1:50 dilution for IHC, 1:1000 dilution for WB), NF-kB (p65) (8242; 1:500 dilution for IHC, 1:1000 dilution for WB), IL-1β (12,242; 1:500 dilution for IF, 1:1000 dilution for WB), β-actin (4970; 1:1000 dilution), were purchased from Cell Signaling Technology (US). Other reagents were purchased from Thermo Fisher, unless otherwise specified.

### TM4 cell culture and PS-MPs exposure

Testicular sertoli cells (TM4 cells) were purchased from Cell Bank of the Chinese Academy of Sciences (Serial, GNM41) and cultured in DMEM/F12 (1:1) medium supplemented with 2.5% fetal bovine serum, 5% horse serum, 1% penicillin/streptomycin in 37 °C and 5% CO_2_. Based on pre-experimental results and previous studies [22], we chose three concentrations of PS-MPs (0.25 mg/mL, 0.5 mg/mL and 1 mg/mL; n = 3) to treat TM4 cells for 24 h. Control group were treated with an equal volume of complete medium.

### PS-MPs exposure in vivo and tissue collection

8-week-old male C57 mice were used for the in vivo study of the toxicity of PS-MPs, All animal experiments were approved by the Animal Ethical Committee of Jilin Agricultural University. Mice were randomly divided into three groups (n = 5 each groups). The mice were housed five per cage and free access to diet and water at room temperature and controlled humidity. The mice in experimental group were treated with 1 μm PS-MPs diluted by ddH_2_O for 4 weeks. The minimum amount of water intake per mouse per day was 6 mL. Hence, the daily intake of PS-MPs was 1 mg/kg (low dose) and 5 mg/kg (high dose) per mouse by conversion. Control mice were given ddH_2_O. After that, the testis tissue was isolated for following experiments.

### The observation of PS-MPs accumulation in TM4 cells

TM4 cells and green fluorescent labelled PS-MPs (1 μm;) were co-cultured in 37 °C and 5% CO_2_ incubator for 24 h. The nuclei were stained by Hoechst 33,258 (Beyotime, C1017). The fluorescence images were observed by confocal laser scanning microscope (CLSM; Olympus FV3000).

### Cell viability assay

MTT assay was applied to determine the cell viability. TM4 cells were grown in 96-well plate. Experimental group were exposed to various concentrations of PS-MPs for 24 h and control group were treated with complete medium. Then both groups cells were starved for 1 h in serum-free medium. After that, MTT solution (1 mg/mL) was added to the plates with 100 µL per well. Next, dimethyl sulfoxide (DMSO) was added to remove crystal after MTT incubation. Optical density (OD) value was measured by microplate reader (Thermo Scientific, Multiskan FC).

### Senescence-associated β-Galactosidase (SA-β-Gal) staining

The number of SA-β-Gal positive cells was a representative biomarker of cellular senescence, Sa-β-gal level was detected by CellEvent Senescence Green kit (Thermo Fisher, C10851) according to manufacture’s instruction. In breif, TM4 cells were seeded in 96-well and exposed to PS-MPs for the indicated concentrations and time points. After washing with phosphate-buffered saline (PBS), the cells were fixed by 2% paraformaldehyde (PFA; Coolaber, SL1830) for 10 min at room temperature. Followed by washing with 1% BSA in PBS to remove PFA, the cells were stained with CellEvent Green Senescence probe for 2 h at 37℃ without CO_2_. The fluorescence images were observed by confocal laser scanning microscope (CLSM; Olympus FV3000) and the fluorescent intensity of SA-β-Gal staining were quantified by Image J software.

### Cell cycle and apoptosis analysis

Cell cycle was determined by propidium (PI) staining (Beyotime, C1052). According to the manufacture’s instruction, after digestion by trypsin, the cells were collected by centrifugation at 1000 ×g for 5 min. The cell samples were resuspended with PBS, the cells were then fixed by 70% ethanol at 4 ℃ for 12 h. After washing, the cells were stained with PI at 37 ℃ in the dark for 30 min. Cell cycle analysis were assessed by FACSCalibur flow cytometer (BD FACSCalibur, C6).

The measurement of apoptosis was performed by Annexin V-FITC/ PI Apoptosis Detection Kit (Beyotime, C1062). According to the manufacture’s instruction, after digestion by trypsin, the cells were harvested by centrifugation at 1000 ×g for 5 min. Then the cells were resuspended with PBS. Subsequently, Annexin V-FITC (200 µL) and PI (10 µL) staining solution was added to each centrifuge tube and incubate for 20 min in the dark at room temperature. Cell samples were detected with FACSCalibur flow cytometer (BD FACSCalibur, C6).

### ROS determination

The detection of ROS level was performed by Reactive Oxygen Species Assay Kit (Beyotime, S0033). The cells were grown in 6-well and detected by DCFH-DA fluorescent probe, which was diluted to 5 µmol/L. After exposed to PS-MPs, TM4 cells were incubated with DCFH-DA for 20 min at 37 ℃. After washing thrice with serum-free culture medium, the fluorescence images were observed by CLSM (Olympus FV3000).

### Sample preparation and western blot analysis

TM4 cells from experimental groups and control groups were lysed by RIPA lysis buffer (Beyotime, P0013B) and centrifuged at 20,000 r/min for 20 min to collect the proteins. The protein concentration was measured by BCA protein concentration determination kit (Beyotime, P0010). The samples (30 µg/lane) were electrophoresed in 10% SDS-PAGE gels and transferred to PVDF membranes (Merk Millipore, IPVH00010). Subsequently, the protein samples were blocked with 5% non-fat powdered milk (Beyotime, P0216) for 1 h. After washing, the membranes were incubated with corresponding primary antibody at 4 °C overnight. After incubation, the samples were washed thrice with TBST and incubated with HRP-conjugated goat anti-rabbit IgG (1:2000 dilution; Proteintech Group, SA00001-2) at room temperature for 1 h. Finally, the membranes were washed with TBST again to reduce non-specific binding. The proteins blot were detected by ECL, and the analysis of grayscale values was performed by ImageJ software.

### Indirect immunofluorescence (IF) assay

After treatment with PS-MPs, TM4 cells were starved for 6 h in serum-free medium. The cells were rinsed with PBS and fixed by PFA (4%). Subsequently, TM4 cells were permeabilized with 0.1% Triton X-100 for 1 h (Beyotime, P0096) and then blocked with 5% bovine serum albumin (BSA) (Beyotime, ST023) for 2 h. After that, the samples were incubated with primary antibody at 4℃ overnight and followed by incubationwith Anti-rabbit IgG (1:1000 dilution; CST, 7074) for 2 h at room temperature (RT). After three final washes with PBS, the samples were observed under CLSM (Olympus FV3000) and quantitative analysis was conducted by using ImageJ software.

### Ca^2+^ signaling detection

The detection of Ca^2+^ signaling level was performed by Fluo-4 Calcium Assay Kit (Beyotime, S1061). Briefly, Fluo-4 AM (500X) was prepared to 2 µL/mL Fluo-4 staining solution according to manufacture’s instruction. The cells grown in 6-well plates were incubated with Fluo-4 staining solution for 30 min at 37℃. After incubation, the cells were washed three times with PBS. The fluorescence images were detected by CLSM (Olympus FV3000) and quantified by Image J software.

### Determination of mitochondrial membrane potential (MMP), mtROS and ATP content

MMP detection was performed by Mito-Tracker Red CMXRos kit (Beyotime, C1049B). After treatment with PS-MPs for 24 h, the cells were incubated with Mito-Tracker Red CMXRos (50 nM) for 20 min at 37^°^C. The cells were then washed thrice with PBS before the detection of red fluorescence intensity by CLSM (Olympus FV3000) and quantified by ImageJ software.

ATP Content Assay Kit (Solarbio, BC0300) was used for ATP content assay. Briefly, the cell were lysed and centrifuged at 12,000 r/min at 4^°^C for 5 min, and supernatant were then collected. The ATP content was determined according the manufacture’s instruction. The samples were measured by a microplate reader (Thermo Scientific, Multiskan FC).

The detection of mtROS was performed by MitoSOX™ Green reagent (Thermo Fisher, M36008). TM4 cells were seeded in 6-well plate and exposed to PS-MPs for 24 h. Followed by washing with PBS, the cells were incubated with 10 µM MitoSOX™ for 20 min at 37^°^C. The fluorescent images were visualized by CLSM (Olympus FV3000) and quantitative analysis by ImageJ software.

### Immunohistochemistry (IHC) assay

The testis tissue embedded in paraffin were cut into slides (5-µm thickness). Sections were deparaffinized and rehydrated.Then the slides were treated with antigen retrieval solution for 20 min, after which the samples were blocked with goat serum at room temperature for 20 min and incubated with primary antibody overnight at 4℃. After washing three times with PBS, the samples was stained with HRP-labeled Goat Anti-Mouse IgG (1:50 dilution; Beyotime, A0216). After rinsing the sections again, the samples were incubated with DAB solution. Then the slides were observed under an inverted microscope (Axio Inspector ZEISS, Germany). Values were analyzed by Image J software (NIH, USA).

### Hematoxylin and eosin (HE) staining

Briefly, after dewaxing and rehydration, the 5-µm thickness testicular tissue sections were stained by hematoxylin and eosin according to the manufacturer’s instructions. The samples were observed by inverted microscope (Olympus, Japan).

### Masson staining

Briefly, after dewaxing and rehydration, the slides were washed with PBS. The samples were then stained by Masson staining kit. The samples were observed by inverted microscope (Olympus, Japan).

### Statistical analysis

All data are presented as the mean ± standard error of mean (SEM). All results were subjected to unpaired Student’ s test or one-way ANOVA, Pearson’s correlation coefficient test with GraphPad Prism 9.5. Data were considered statistically significant when *p < 0.05, **p < 0.01, ***p < 0.001.

## Results

### The effect of PS-MPs exposure on testicular structures

**Fig. 1:**
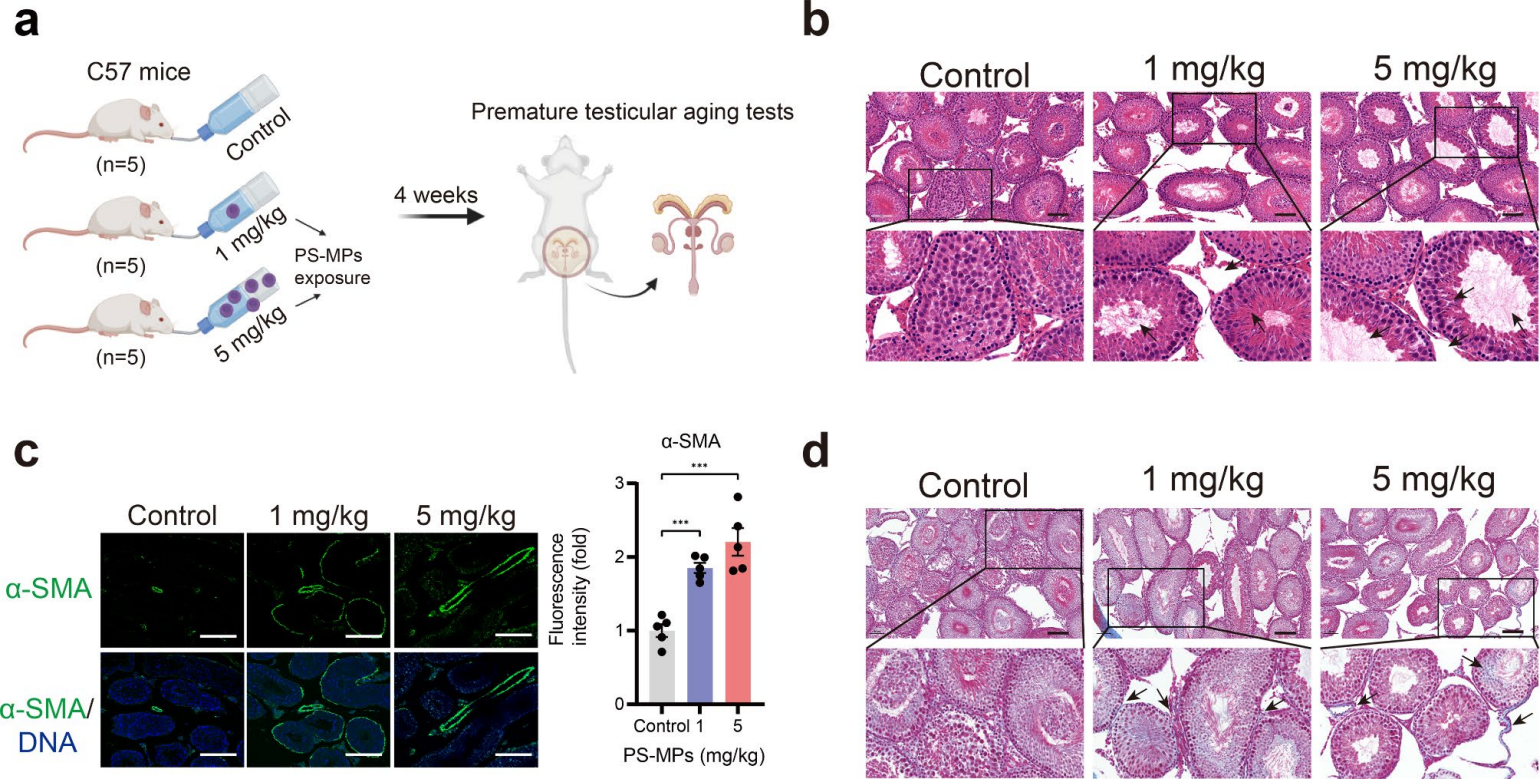
The toxicity of PS-MPs to the testicular tissue. **a** Schematic diagram of in vivo experimental design. **b** HE staining showing pathological changes of the testicular tissue after PS-MPs exposure. **c** IFA images showing comparative and quantitative analysis of α-SMA expression levels in the testis between control mice and PS-MPs exposure mice. **d** Masson staining showing the extent of fibrosis in the testicular tissue after PS-MPs exposure. Scale bars: 100 μm in **b** and **c**; 30 μm in **d**

To explore the effect of PS-MPs exposure on premature testicular aging in vivo, we treated normal 8-week-old C57 WT mice with control (ddH_2_O) or PS-MPs (1 mg/kg or 5 mg/kg via drinking ddH_2_O) (n = 5) for 4 weeks (Fig. 1a). To assess the toxicity of PS-MPs on mice testis, we checked the alteration in morphology of testicular tissue after exposure to PS-MPs for 4 weeks. In control mice, various spermatogenic cells (spermatogonium, spermatocyte, spermatid) were abundant and tightly arranged in the testis. A great quantity of spermatozoa were equally distributed on the surface of the lumen via HE staining. However, in the PS-MPs exposure group, spermatogenic cells were fallen off and loosely arranged. The number of spermatozoa was declined and blank cavities appeared in the testis (Fig. 1b). Further, we wonder whether PS-MPs exposure causes testicular tissue fibrosis. As IFA data shown, the expression level of alpha -smooth muscle actin (α-SMA) was increased significantly (Fig. 1c). Furthermore, Masson staining results indicated that testicular tissue fibrosis was highly pronounced after exposure to PS-MPs compared to control mice (Fig. 1d).

### PS-MPs exposure triggers premature testicular aging

**Fig. 2:**
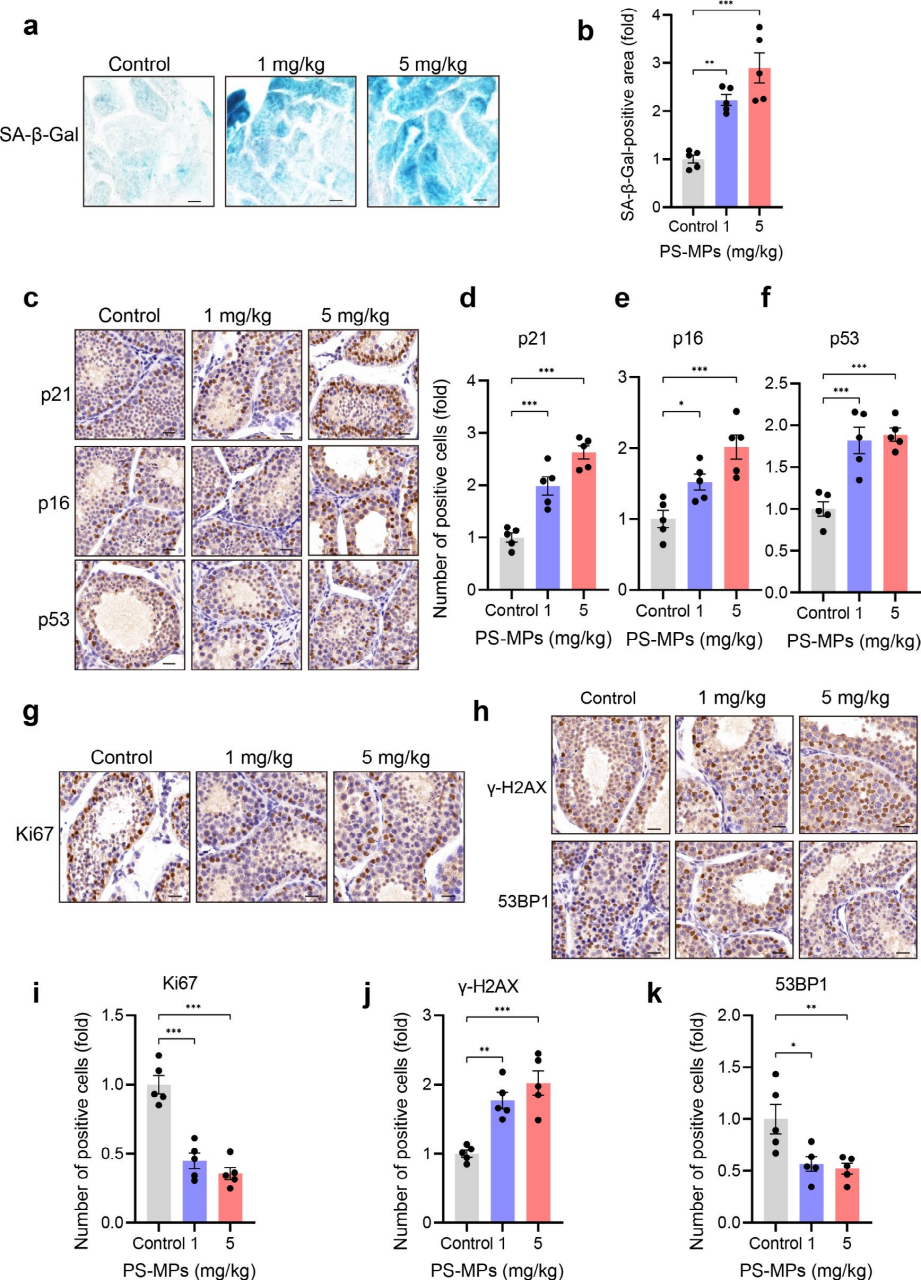
PS-MPs exposure triggers premature testicular aging. **a** SA-β-Gal staining of testicular tissue. **b** Quantitative analysis of SA-β-Gal staining as presented in **a**. **c** Representative IHA images of p21, p16 and p53 in the testis from control and PS-MPs exposure mice. **d-f** Quantification of p21, p16, p53-positive cells as shown in **c**. **g, h** Representative IHA images depicting Ki67, γ-H2AX and 53BP1 in the testis following PS-MPs treatment. **i-k** Quantification of Ki67, γ-H2AX and 53BP1-positive cells as described in **g**, **i**. Scale bars: 50 μm (all panels)

As Fig. 2 shown, following PS-MPs exposure for 4 weeks, we tested the effect of PS-MPs on the premature aging in the testis by estimating the expression level of spermatogenic cells. The SA-β-Gal-positive area was increased significantly in PS-MPs-exposed mice compared to control mice (Fig. 2a, b). Consistently, PS-MPs exposure elevated the expression of senescence marker (including p21, p16, and p53) in testicular tissue, as shown by increased number of positive spermatogenic cells (Fig. 2c-f). Next, we detected the markers of DNA damage response—histone H2AX phosphorylation (γ-H2AX) and p53-binding protein 1 (53BP1). Our results showed the remarkable enhanced γ-H2AX expression level in PS-MPs-treated mice. The expression level of 53BP1, a chromatin-binding protein regulating the repair of DNA double-strand breaks, was measured. By contrast to γ-H2AX, the number of 53BP1 positive cells was lower after PS-MPs treatment (Fig. 2h, j, k). Figure 2 g, i showed a decreased expression level of Ki67 in the testis after PS-MPs treatment, indicating that exposure to PS-MPs reduced the ability of testicular cell proliferation. The merged results suggested that PS-MPs exposure triggers premature testicular aging.

### PS-MPs exposure activates oxidative stress in the testis

**Fig. 3:**
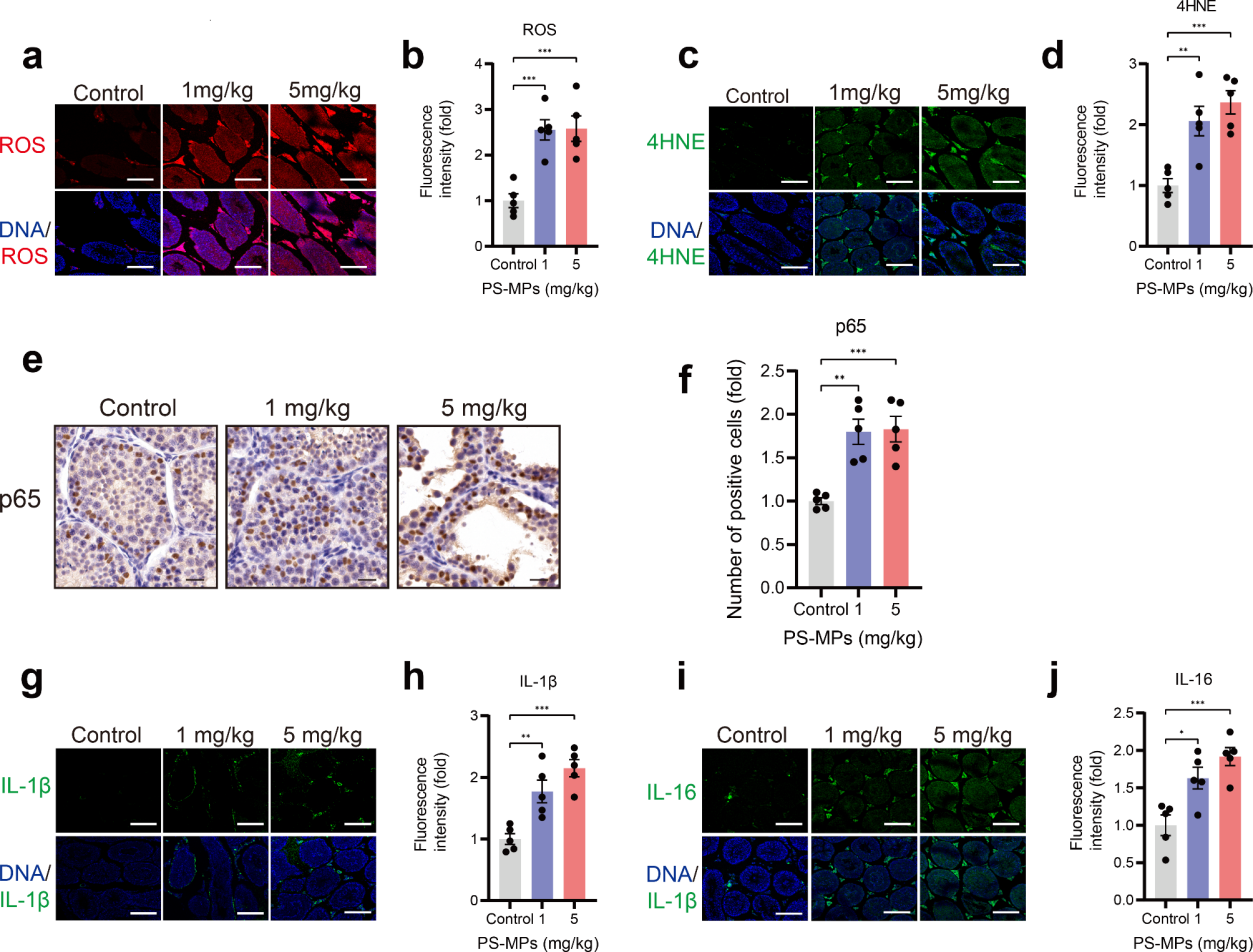
The effect of PS-MPs exposure on oxidative stress and inflammatory response in vivo. **a** Determination of ROS content by CLSM in control and PS-MPs exposure group. **b** Quantitative analysis of ROS described in **a**. **c** Representative IFA images of 4-HNE in the testis from three groups. **d** Comparative quantification of 4-HNE expression level mentioned in **c**. **e** IHA images showing the measurement of p65 in the testis treated with ddH_2_O and PS-MPs. **f** Quantification of p65-positive cells described in **e**. **g-j** Representative fluorescent images showing the detection of IL-1β and IL-16 and quantitative analysis of them. Scale bars: 50 μm in **e**; 30 μm in  **a**, **c**, **g** and **i**

We next queried whether PS-MPs exposure can induce oxidative stress in vivo. As Fig. 3a-d presented, IF was performed to examine ROS and 4-Hydroxynonenal (4-HNE) content in spermatogenic cells after PS-MPs exposure, and the results indicated that both ROS and 4-HNE levels were remarkably higher in the testis treated by PS-MPs, suggesting that exposure to PS-MPs can activate oxidative stress in the testis.

### PS-MPs exposure induces inflammatory response in the testis

To further investigate the toxicity of PS-MPs to male reproductive system, we detected the inflammation response after PS-MPs treatment. We measured the level of p65 (one of the major subunit of the NF-κB complex) to estimate the activity of NF-κB. The IHC results revealed increased p65 level in PS-MPs-treated testis. (Fig. 3e, f). Activated NF-κB in turn induces the elevated expression of downstream pro-inflammatory factors (IL-1β and IL-16) (Fig. 3g-j), which was defined as senescence-associated secretory phenotype (SASP).

### Internalization of PS-MPs

**Fig. 4:**
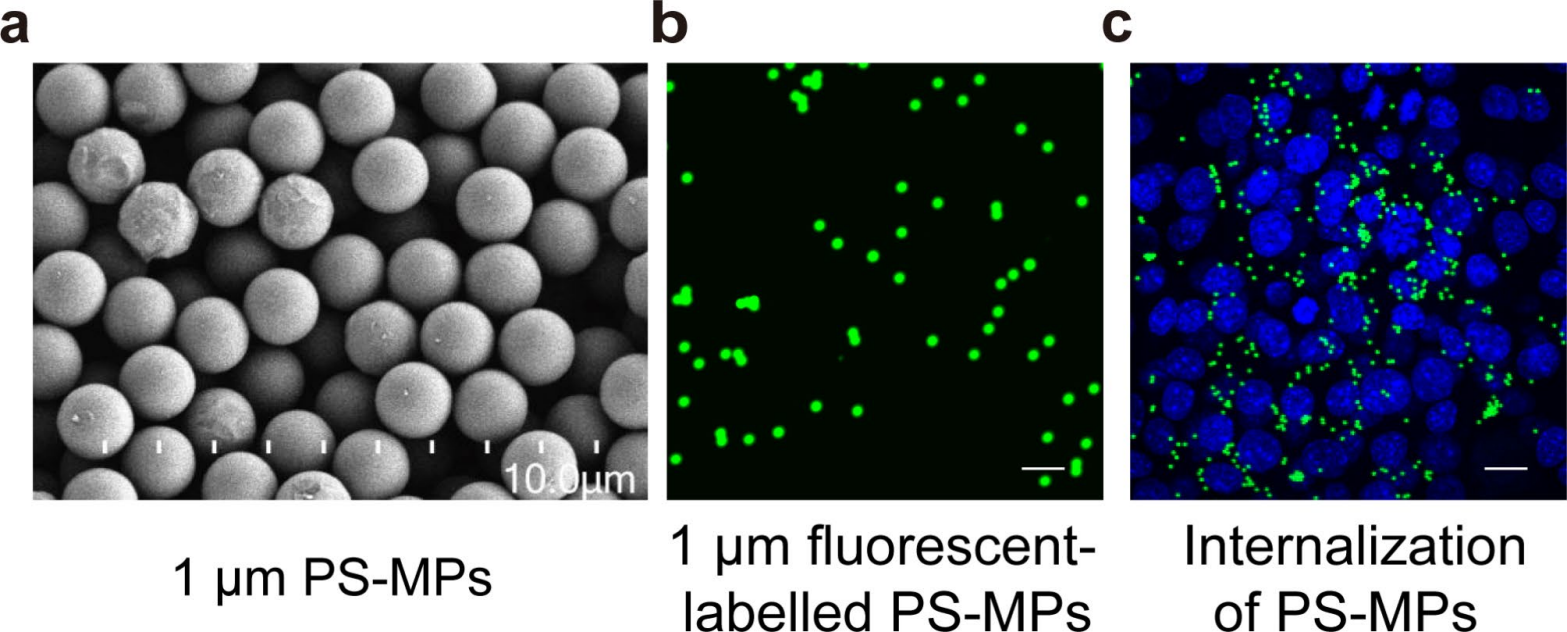
Characterization and internalization of PS-MPs. **a** PS-MPs with 1 μm particle size observed under scanning electron microscope. **b** Fluorescent PS-MPs with 1 μm particle size observed under CLSM. **c** Internalization of PS-MPs in TM4 cells. Scale bars: 30 μm in b and 50 μm in c

We used scanning electron microscope to observe the features of 1 μm PS-MPs, which were exhibited as uniformly spherical in size (Fig. 4a). Fluorescent PS-MPs were identified by CLSM (Fig. 4b), and the observation of PS-MPs internalization into TM4 cells was investigated using fluorescent PS-MPs. As shown in Fig. 4c, after fluorescent PS-MPs administration for 24 h, a great amount of PS-MPs accumulated in the cells, indicating that PS-MPs (1 μm) can internalize into TM4 cells.

### PS-MPs exposure triggers premature senescence TM4

**Fig. 5:**
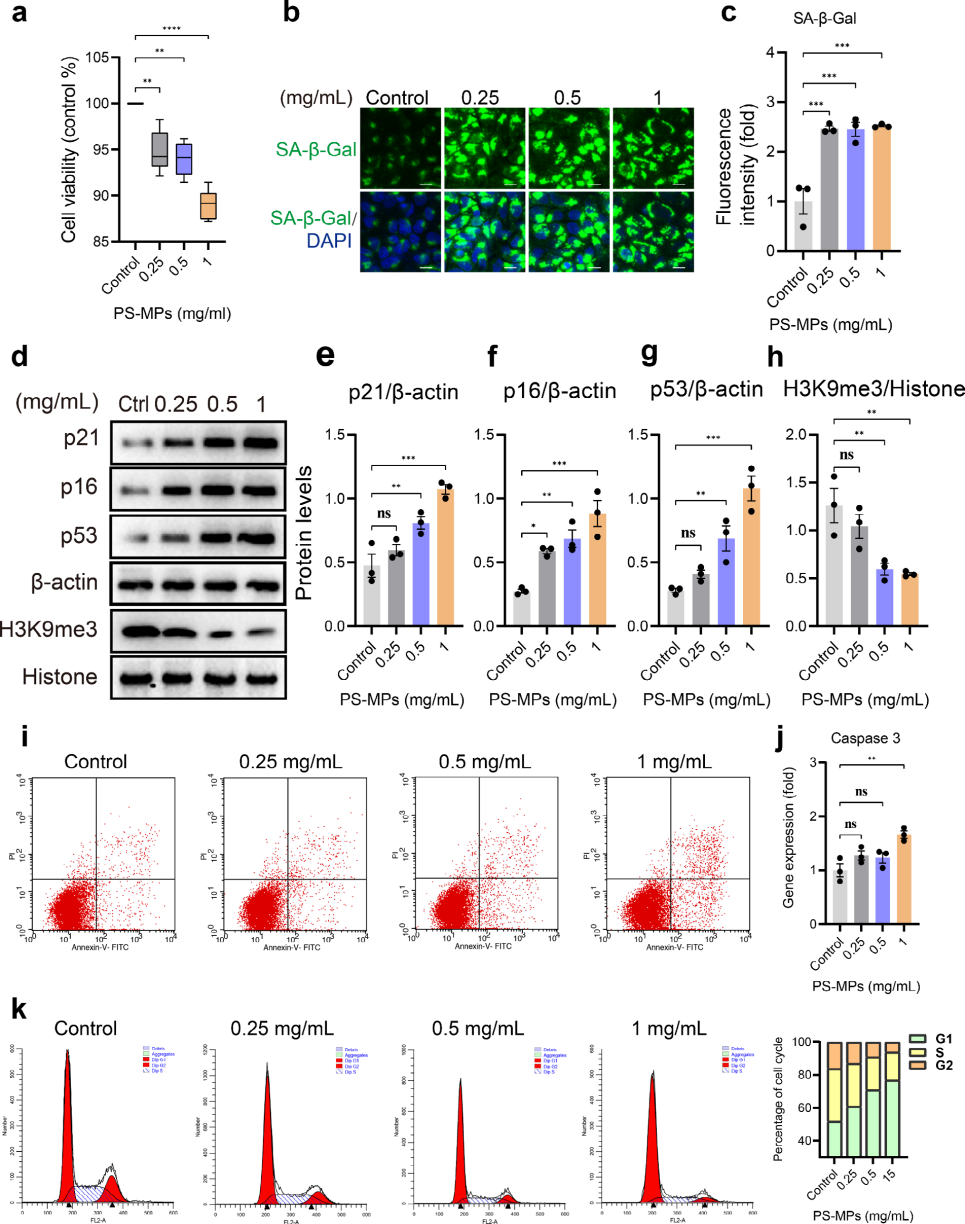
PS-MPs exposure caused premature senescence of TM4 cells. **a** Box plot showing cell viability after PS-MPs exposure for 24 h. **b** Representative fluorescent images of SA-β-Gal staining in TM4 cells. **c** Quantitative analysis of SA-β-Gal staining as presented in **b**. **d** Western blot of p21, p16, p53 and H3K9me3 in TM4 cells treated with control and PS-MPs. **e-h** Quantification of protein levels of p21, p16, p53 and H3K9me3 described in **d**. β-actin and Histone considered as loading control. **i**, **k** Flow cytometry showing apoptotic cells and percentage of cell cycle. **j** qPCR analysis of Caspase 3. The representative images were selected from three independent and replicated experiments (n = 3). Scale bars: 30 μm

Figure 5a showed that cell viability was decreased significantly after PS-MPs treatment. β-galactosidase activity as a classical marker of cellular senescence showed that the number of SA-β-gal-positive cells remarkably increased in PS-MPs group (Fig. 5b, c). We further detected the greatly enhanced expression of p53, p21 and p16 compared to the control group (Fig. 5d-g). As presented by Fig. 5d, h, PS-MPs exposure decreased the expression of trimethylated histone H3 Lys9 (H3K9me3), a marker of senescence-associated heterochromatin foci (SAHF) [23]. In addition, cell cycle analysis indicated that PS-MPs exposure led to a marked arrest of cells in G0-G1 phase in a concentration-dependent manner (Fig. 5k). Additionally, we assessed the effect of PS-MPs on TM4 cell apoptosis. The results illustrated that PS-MPs exposure with 0.25 mg/mL and 0.5 mg/mL did not induce significant cell apoptosis. However, the administration of 1 mg/mL PS-MPs was accompanied by a significant increase in apoptosis rate (Fig. 5i, j). The merged results demonstrated that exposure to PS-MPs triggers premature senescence in TM4 cells.

### PS-MPs exposure induces oxidative stress in TM4 cells

**Fig. 6:**
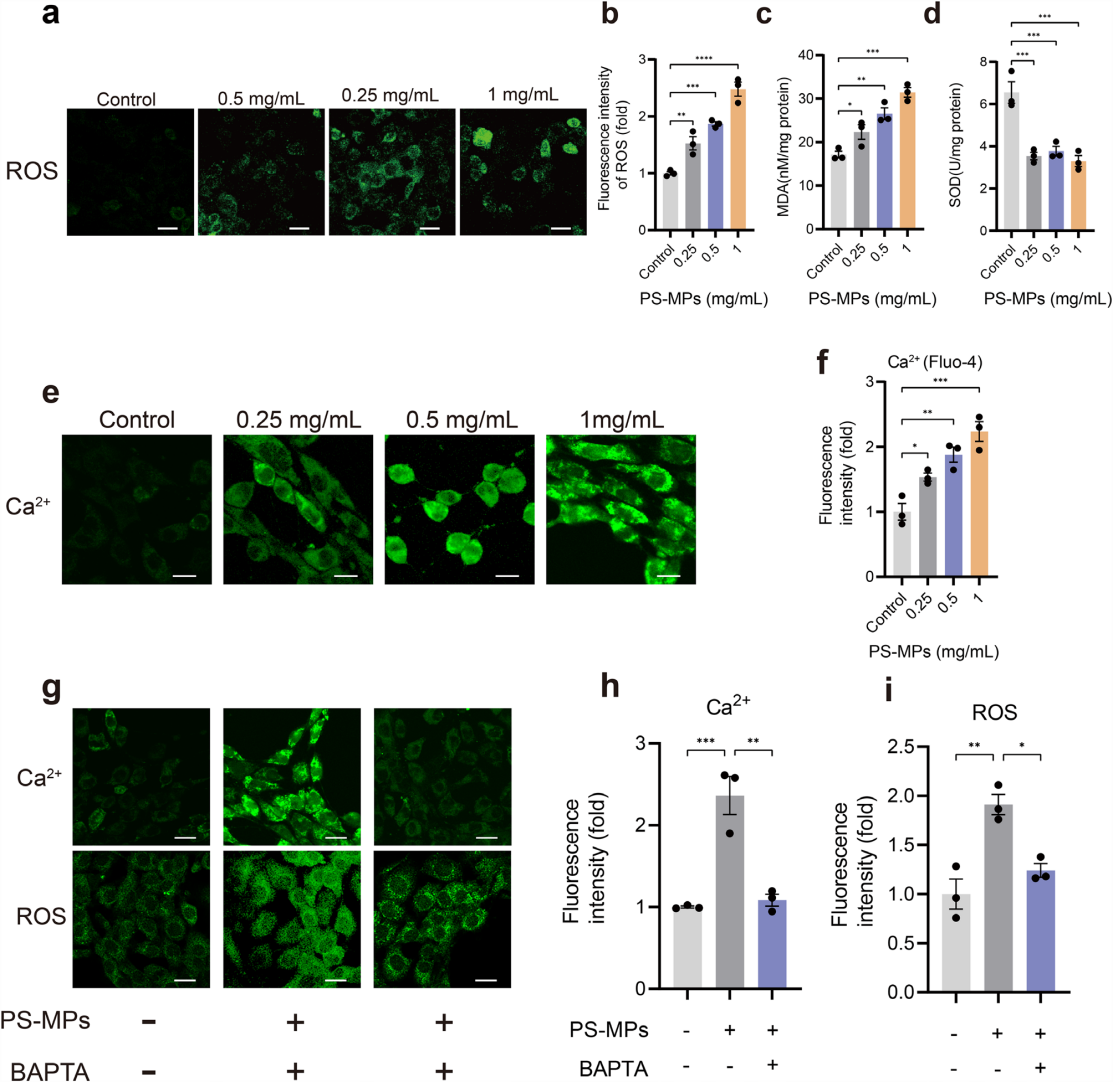
The effect of PS-MPs exposure on oxidative stress and Ca^2+^ signaling in TM4 cells. **a** Measurement of ROS levels in TM4 cells by CLSM. **b** Quantitative analysis of ROS presented in **a**. **c**, **d** Comparative statistics of MDA and SOD content between control group and PS-MPs exposure groups. **e** Representative fluorescent images showing Ca^2+^ signaling after PS-MPs exposure. **f** Quantification of fluorescent intensity of Ca^2+^ signaling as presented in **e**. **g** Representative fluorescent images of Ca^2+^ signaling and ROS levels in TM4 cells treated with control, PS-MPs and PS-MPs + BAPTA. **h**, **i** Comparative quantification of fluorescent intensity of Ca^2+^ signaling (H) and ROS (I) as presented in **g**. The representative images were selected from three independent and replicated experiments (n = 3). Scale bars: 30 μm

Oxidative stress is one of the driving factors of cellular senescence. To explore whether PS-MPs can induce oxidative stress in TM4 cells, we measured ROS level by CLSM. The results revealed that ROS level were enhanced significantly in TM4 cells in response to PS-MPs exposure (Fig. 6a, b). Furthermore, we confirmed the increased MDA content and reduced SOD content in PS-MPs-exposed TM4 cells (Fig. 6c, d).

### PS-MPs exposure causes Ca^2+^ overload in the mitochondria

Previous studies showed that Ca^2+^ overload in the mitochondria is commonly accompanied by oxidative stress [24]. Thus, we explored whether PS-MPs-caused oxidative stress was induced by Ca^2+^ overload in the mitochondria. To this end, we carried out the corresponding experiments. As shown by Fig. 6e, f, PS-MPs exposure led to a marked increase in Ca^2+^ signaling. Subsequently, BAPTA, a Ca^2+^ chelator, was employed to treat TM4 cells exposed to PS-MPs (0.5 mg/mL). We found that the reduction in Ca^2+^ was accompanied by a significant reduction in ROS levels (Fig. 6g-i), indicating that PS-MPs-induced oxidative stress was caused by Ca^2+^ overload in the mitochondria.

### PS-MPs exposure causes mitochondrial dyshomeostasis

**Fig. 7:**
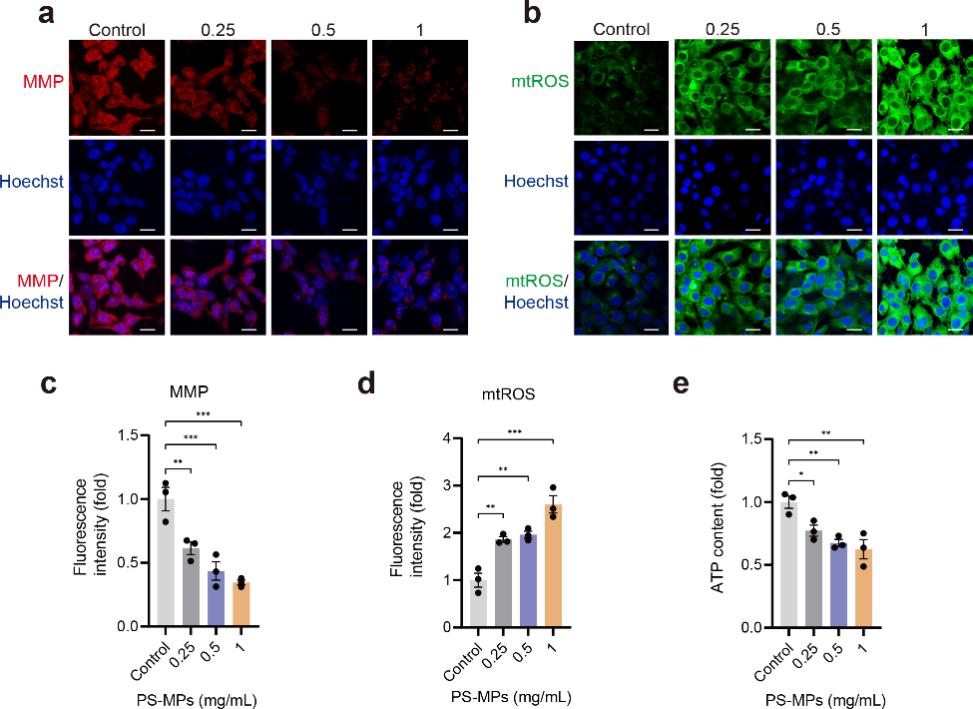
The effect of PS-MPs exposure on mitochondrial homeostasis. **a**, **b** Representative fluorescent images showing the comparison of MMP and mtROS levels between control cells and PS-MPs cells. **c-e** Comparative statistics of MMP, mtROS described in **a**, **b** and ATP content. The representative images were selected from three independent and replicated experiments (n = 3). Scale bars: 30 μm

Mitochondrial Ca^2+^ dysregulation is a major factor inducting mitochondrial damage and dyshomeostasis. Thus, following the discovery of PS-MPs-induced mitochondrial calcium overload, we measured mitochondrial membrane potential (MMP). The experimental results showed that MMP level were significantly decreased after treatment with PS-MPs (Fig. 7a, c). We next assayed ATP content to further evaluate the mitochondrial dysfunction. ATP content was greatly reduced after exposure to PS-MPs (Fig. 7e).

Reduced MMP and mtROS overproduction interact with each other in damaged and dysregulated mitochondria [25]. Zorov et al. reviewed that decreased MMP is accompanied by mtROS overproduction through electron transfer chain, and that mtROS release can induce the loss of MMP [26]. Therefore, we assessed mitochondrial superoxide generation after PS-MPs exposure. PS-MPs exposure increased mtROS level remarkably (Fig. 7b, d), suggesting that PS-MPs-induced elevation of intracellular ROS is probably resulted from mtROS leakage.

### PS-MPs exposure activates NF-κB signaling pathway

**Fig. 8:**
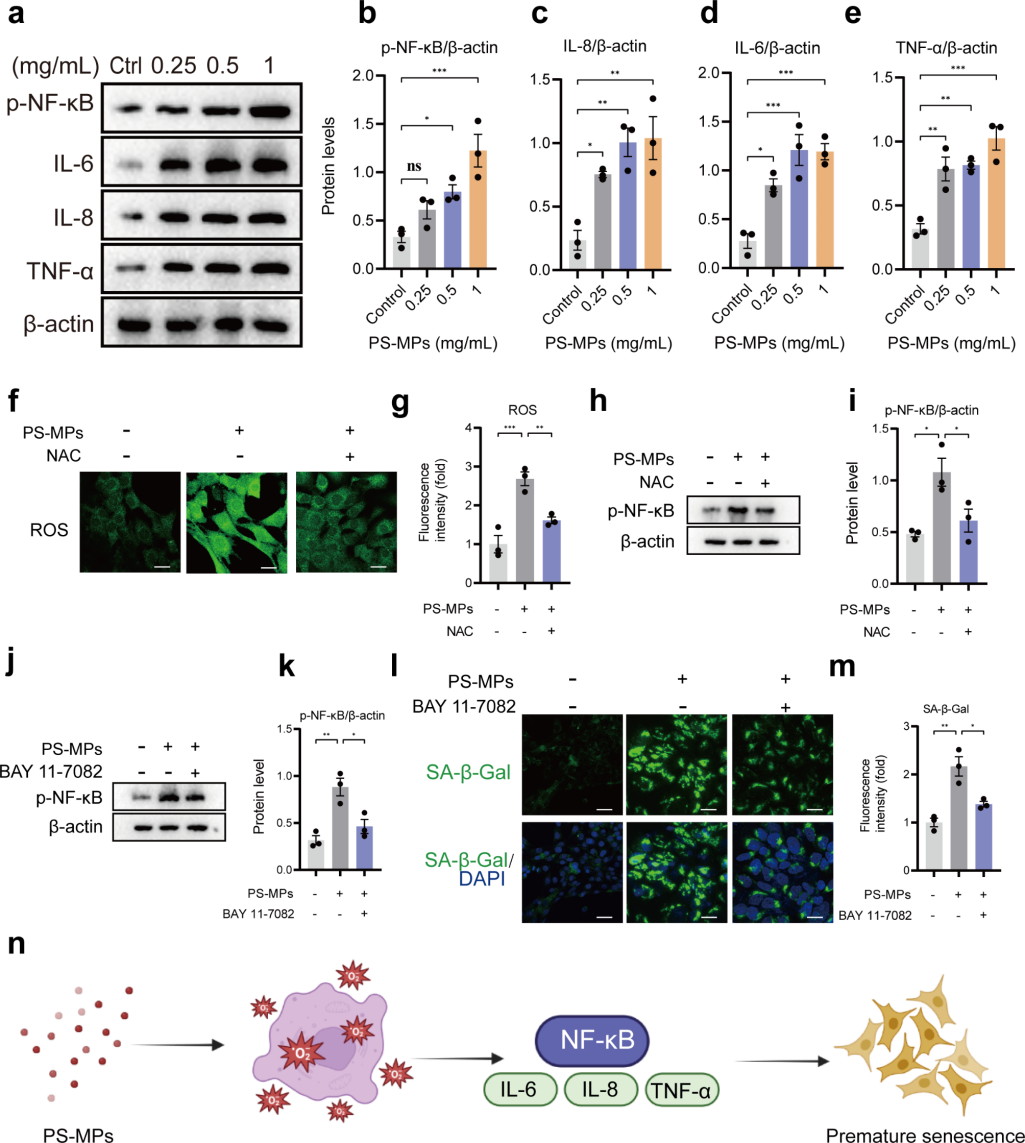
The effect of PS-MPs exposure on inflammatory response. **a** Western blot of NF-κB, IL-6, IL-8 and TNF-α in TM4 cells treated with control and PS-MPs. **b-e** Quantification of protein levels of NF-κB, IL-6, IL-8 and TNF-α described in **a**. β-actin considered as loading control. **f** Detection of ROS levels in TM4 cells after treated with PS-MPs and PS-MPs + NAC. **g** Quantitative analysis of fluorescent intensity in **f**. **h**, **j** Western blot showing the expression of p-NF-κB in TM4 cells after treated with PS-MPs and PS-MPs + NAC (H) or PS-MPs + BAY 11-7082 **j**. **i** Quantification of protein levels of p-NF-κB described in **h**, **j**. **l** Representative fluorescent images showing SA-β-Gal activity treated with PS-MPs and PS-MPs + BAY 11-7082. **m** Quantitative analysis of fluorescent intensity of SA-β-Gal described in **l**. **n** Schematic diagram of PS-MPs causing premature TM4 cellular senescence through ROS and NF-κB mediation. The representative images were selected from three independent and replicated experiments (n = 3). Scale bars: 30 μm

It has been reported that the downstream effects of ROS generated in the mitochondria usually activate NF-κB signaling pathway [27]. Compared to control group, exposure to PS-MPs resulted in higher level of inflammation, as evidenced by significant up-regulation of p-NF-κB (Fig. 8a, b). Then we applied N-Acetyl-L-cysteine (NAC) (1µM) to eliminate ROS and the results showed that NAC obviously inhibited ROS production in PS-MPs exposure group (Fig. 8f, g). As expected, the expression of p-NF-κB induced by PS-MPs was also suppressed after NAC treatment (Fig. 8h, i), demonstrating that PS-MPs-induced p-NF-κB activation is modulated by ROS. In addition, western blot analysis showed that the expression levels of pro-inflammatory factors (IL-6, IL-8 and TNF-α) were dramatically up-regulated in PS-MPs exposure group compared to control group (Fig. 8a and c-e).

Further, whether NF-κB is one of causes of PS-MPs-induced cellular senescence was investigated. p-NF-κB was down-regulated greatly after administration of 10 µM BAY 11-7082 (NF-κB inhibitor) to TM4 cells exposed to PS-MPs (Fig. 8j, k). Meanwhile, the fluorescent intensity of SA-β-Gal reduced significantly, suggesting that PS-MPs-induced TM4 cellular senescence was through NF-κB mediation (at least partially) (Fig. 8l, m). Figure 8n shows a schematic diagram of PS-MPs causing premature cellular senescence via ROS/NF-κB signaling pathway.

### PS-MPs exposure impairs mitophagy

**Fig. 9:**
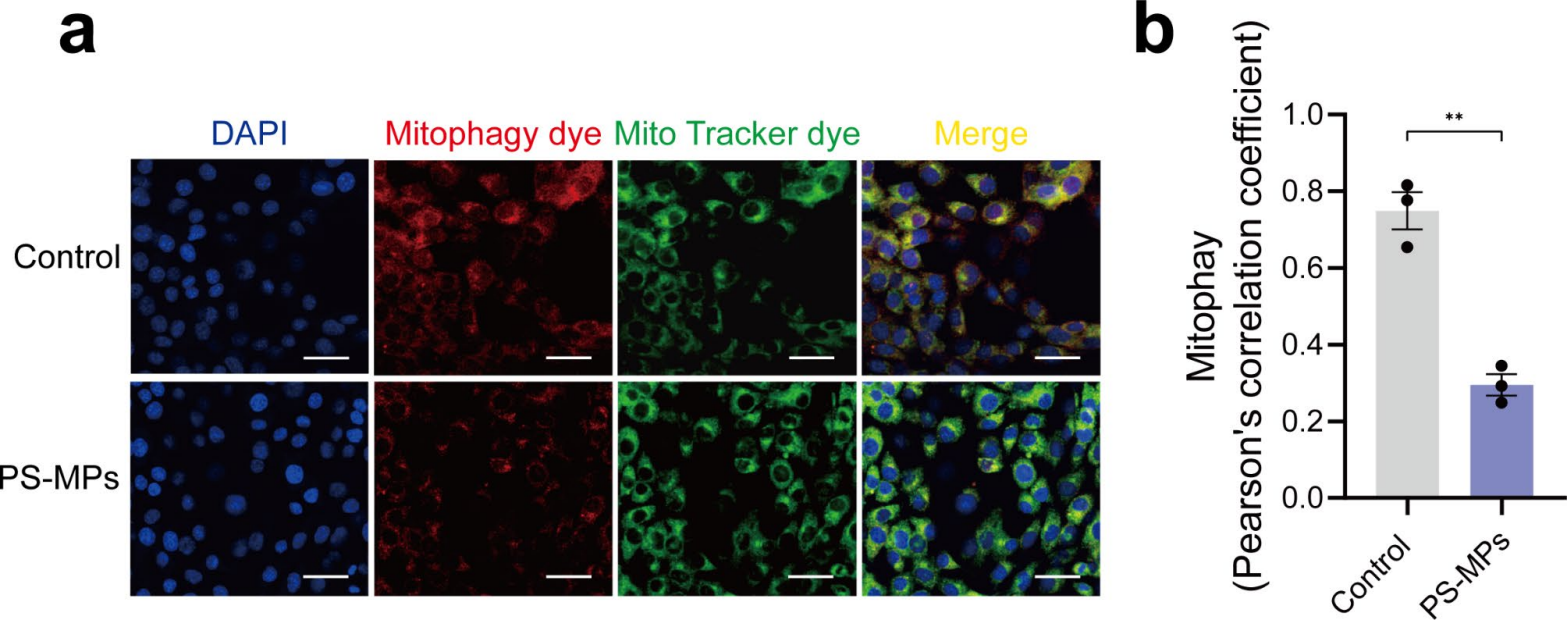
The effect of PS-MPs exposure on mitophagy. **a** Representative images showing the levels of mitophagy in control group and PS-MPs exposure group. **b** Comparative quantification of mitophagy levels mentioned in **a**. Quantitative analysis was based on the Pearson’s correlation coefficient to analyze the co-localization level. The representative images were selected from three independent and replicated experiments (n = 3). Scale bars: 30 μm

Autophagy is an essential biological process for cells to maintain physiological functions. Autophagy inhibition is associated with many degenerative disease, such as Parkinson’s disease and Alzheimer’s disease [28]. Autophagy is a process of “self-eating” that cytosolic components are engulfed by autophagic vesicles, fused and degraded by lysosomes [29]. Thus, the effect of PS-MPs exposure on mitophagy was measured. Following the treatment of PS-MPs (0.5 mg/mL), mitophagy was inhibited significantly compared to the control group (Fig. 9a, b), leading to a constant release of mtROS from damaged mitochondria to trigger cellular senescence.

## Discussion

Plastic waste ends up in landfills or in the oceans due to indiscriminate disposal and mismanagement. Plastic waste is hard to degrade, hence, it pollutes marine and soil severe for a prolonged duration. MPs pollution has threatened animals and humans health, such as liver fibrosis and disruption of intestinal barrier [12, 13]. However, the effect of MPs exposure on premature testicular aging has not been reported. López-Otín et al. summarized the hallmarks of aging. They comprise cellular senescence, genomic instability, telomere attrition, epigenetic alterations, loss of proteostasis, deregulated nutrient sensing, mitochondrial dysfunction, stem cell exhaustion, and altered intercellular communication [30]. Cellular senescence is defined as abnormal mitosis due to cell cycle arrest, and is usually manifested as telomere shortening, DNA damage, enlargement of cell volume, elevated NF-κB signaling pathway, occurrence of heterochromatin foci. Senescent cells can be identified by particular biomarkers, such as β-galactosidase activity, p53-governed cyclin-dependent kinase inhibitors (p21 and p16), but there is currently no single gold-standard biomarker [31]. Of note, we found that PS-MPs exposure led to premature testicular aging and premature senescence in TM4 cells. Most elderly people have chronic inflammation. Presence of inflammation in the human body was primarily featured by elevated expression of inflammatory markers in the blood. Chronic inflammation is a risk factor for cardiovascular diseases, obesity, atherosclerosis and depression [32]. Therefore, whether PS-MPs-triggered premature aging is mediated by NF-κB signaling pathway was investigated in this work. In prematurely aged testicular tissue and prematurely senescent cells induced by PS-MPs, the expression levels of p65/NF-κB was increased significantly. Additionally, PS-MPs-caused cellular senescence was alleviated obviously by inhibiting NF-κB signaling pathway in TM4 cells, indicating that premature testicular aging induced by PS-MPs is mediated by NF-κB signaling pathway. In the downstream effects of NF-κB, senescent cells displayed senescence-associated secretory phenotype (SASP) that secrete multiple pro-inflammation factors, e.g. IL-6, IL-8 and TNF-α, which was observed in our study as well. The merged results are consistent with previous studies [33, 34].

Furthermore, we explored the upstream mechanism of PS-MPs-induced inflammatory response. We found that oxidative stress level was enhanced significantly. Inhibition of ROS down-regulated p-NF-κB signaling, suggesting that PS-MPs-induced NF-κB activation is regulated by oxidative stress. It has been widely reported that PS-MPs can induce oxidative stress [15], however, where does ROS originated is the next part of our investigation. Various stimuli like environmental toxins contribute to mitochondrial damage and dyshomeostasis. mtROS overproduction is happened in dysregulated mitochondria. Mitochondrial permeability transition pore (mPTP) or inner membrane anion channel (IMAC) will be opened when the accumulation of mtROS reaches a threshold [35–37]. The elevated level of ROS in the cytoplasm in turn activates mtROS in the adjacent mitochondria, and this mitochondria-mitochondria interaction forms a positive-feedback mechanism contributing to high ROS level, which is called mitochondrial ROS-induced ROS release (RIRR) phenomenon [38]. Our results showed that exposure to PS-MPs caused mitochondria damage and dyshomeostasis, as demonstrated by reduced MMP, ATP content and increased mtROS. Similarly, mitochondria dyshomeostasis has also been found in RBL-2H3 cells exposed to PS-MPs [14]. Numerous published studies support mitochondrial destabilization and mitochondrial oxidant being a key factor in senescence/aging process. The mtROS bursts triggered cellular senescence via activating p53, p21, p16 signaling molecular expression in senescent cells [39–41]. In addition, mitochondrial DNA (mtDNA) in the dysregulated mitochondrial leaks into the cytoplasm through the opening mPTP. Excessive mtDNA that releases into cytoplasm from mitochondria is recognized by cyclic GMP-AMP synthase-stimulator of interferon genes (cGAS-STING) signaling pathway, which then mediates the cellular senescence [42].

Ca^2+^ engaged in the aging process has been reported. Lu and coworkers found the obvious alterations of calcium-related genes in aged human brain [43]. Chelation of Ca^2+^ can alleviate hEMSCs senescence [44]. Wiel and coworkers indicated that Ca^2+^ overload in the mitochondrial decreased MMP and enhanced ROS [45], which is similar with our observations. Our results showed that Ca^2+^ concentration was significantly increased in TM4 cells, and that PS-MPs-induced oxidative stress was regulated by Ca^2+^. The interaction between ROS and Ca^2+^ is involved in a variety of neurodegenerative diseases such as Parkinson’s disease (PD), Alzheimer’s disease (AD) [46]. Elevated Ca^2+^ level in senescent cells might be a consequence of Ca^2+^ release from the endoplasmic reticulum (ER) to mitochondrial. Knockout of inositol 1,4,5-triphosphate receptor (IP3R) (ER calcium release channels) and mitochondrial calcium uniporter (MCU) can attenuate senescence [45]. Additionally, ER–mitochondria contact sites (ERMCSs) which was mediated by mitoguardin (an outer mitochondrial membrane) and vesicle-associated membrane protein (VAMP)-associated protein (Vap33) (an ER protein) facilitates Ca^2+^ transport between ER and mitochondrial [47, 48]. Notably, EMRCSs is involved in age-related disease [49].

Mitophagy is considered as a protective mechanism against sustained cellular damage. Mitophagy involved in anti-aging process has been reported. For example, NAD^+^ alleviates cellular senescence by inducing mitophagy [50]. Once mitophagy is impaired, dysregulated mitochondrial will release ROS continuously, leading to a constant accumulation of senescent cells [51]. Fang et al. observed compromised mitophagy in the hippocampus of AD patients, and mitophagy improves cognitive impairment [52]. Consistently, mitophagy was impaired in PS-MPs-treated TM4 cell in the current work. Mitophagy is mainly mediated by parkin RBR E3 ubiquitin protein ligase (PRNK)-dependent and -independent pathways, and PRKN-dependent mitophagy is mainly mediated by the PINK1. In senescent cells, MMP collapse leads to accumulation of uncleaved PINK1 on outer mitochondrial membranes, allowing it recruit PRKN in the cytoplasm to amplify mitophagy signals [53]. In addition, PRKN-mediated mitophagy suppresses ROS production [54]. Thus, combined with the above data, we hypothesize that PS-MPs inhibit mitophagy by suppressing the PRKN pathway, which is the direction of our further exploration.

Taken together, our study further sheds light on the toxicity of microplastics to the male reproductive system. Combined with earlier studies, microplastics can cause cell death and organism damage in multiple pathways. Plastic pollution is therefore a global problem that needs to be addressed urgently.

## Conclusion

**Fig. 10:**
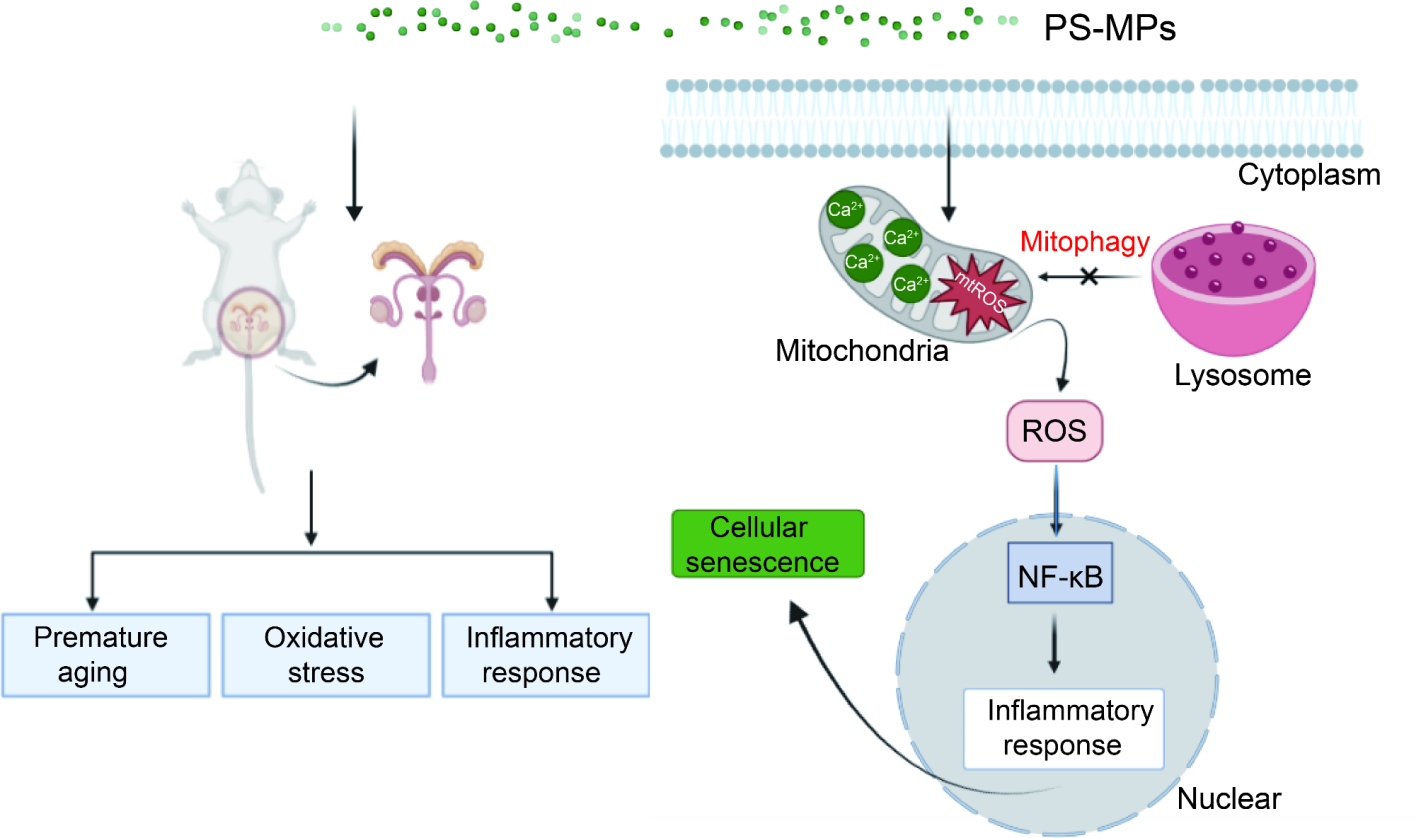
Schematic diagram of molecular mechanism by which PS-MPs exposure causes premature testicular aging

In summary, exposure to PS-MPs triggers premature testicular aging through mediating Ca^2+^/ROS/NF-κB signaling axis. PS-MPs exposure also inhibits mitophagy, leading to the continuous accumulation of senescent TM4 cells (Fig. 10).

### Electronic supplementary material

Below is the link to the electronic supplementary material.

Supplementary Material 1

Supplementary Material 2

Supplementary Material 3

## Data Availability

The datasets used and/or analyzed during the current study are available from the corresponding author on reasonable request.
