# Supplementary material for: Long-term exposure to polystyrene microplastics triggers premature testicular aging
Source: Part Fibre Toxicol. 2023 Aug 28;20:35. doi: 10.1186/s12989-023-00546-6 (PMC10463354; doi:10.1186/s12989-023-00546-6)
Supplement: Supplementary file 1 — Supplementary Material 1 [file 12989_2023_546_MOESM1_ESM.pdf]

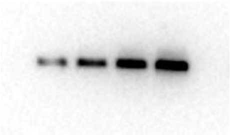

Fig.5d (p21)

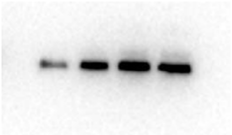

Fig.5d (p16)

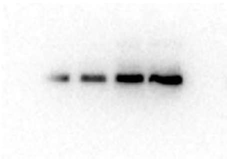

Fig.5d (p53)

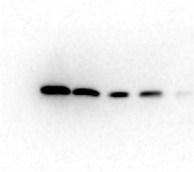

Fig.5d (H3K9me3)

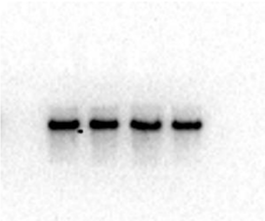

Fig.5d (Histone)

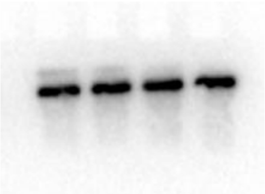

Fig.5d (β-actin)

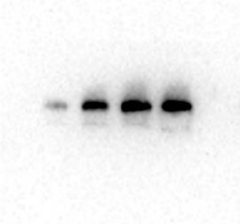

Fig.8a (IL-6)

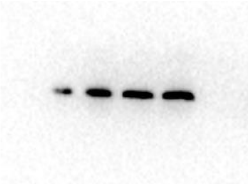

Fig.8a (IL-6)

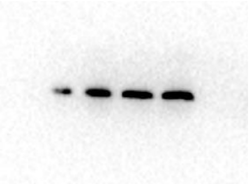

Fig.8a (IL-8)

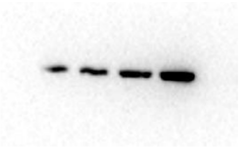

Fig.8a (NF-κB)

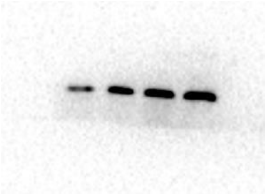

Fig.8a (TNFα)

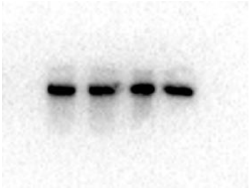

Fig.8a (β-actin)

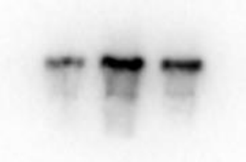

Fig.8h (p-NF-κB)

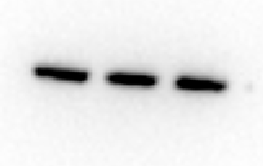

Fig.8h (β-actin)

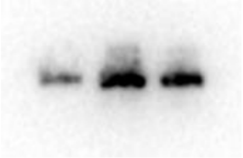

Fig.8j (p-NF-κB)

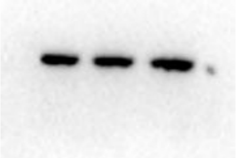

Fig.8j (β-actin)

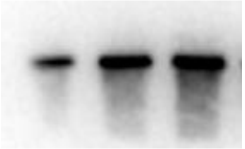

Supplementary Fig.2a (p21)

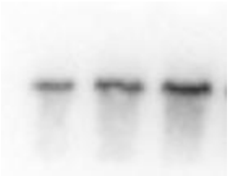

Supplementary Fig.2a (p16)

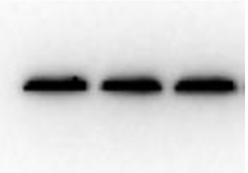

Supplementary Fig.2a (β-actin)

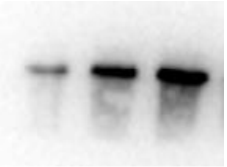

Supplementary Fig.2b (MPO)

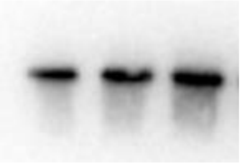

Supplementary Fig.2b (4-HNE)

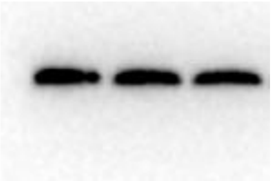

Supplementary Fig.2b (β-actin)

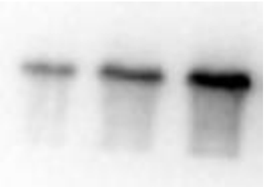

Supplementary Fig.2c (IL-6)

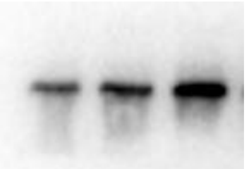

Supplementary Fig.2c (IL-1β)

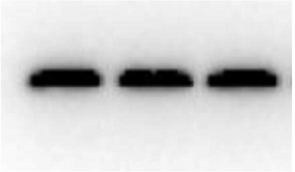

Supplementary Fig.2c (β-actin)
