# Supplementary material for: Long-term exposure to polystyrene microplastics triggers premature testicular aging
Source: Part Fibre Toxicol. 2023 Aug 28;20:35. doi: 10.1186/s12989-023-00546-6 (PMC10463354; doi:10.1186/s12989-023-00546-6)
Supplement: Supplementary file 2 — Supplementary Material 2 [file 12989_2023_546_MOESM2_ESM.pdf]

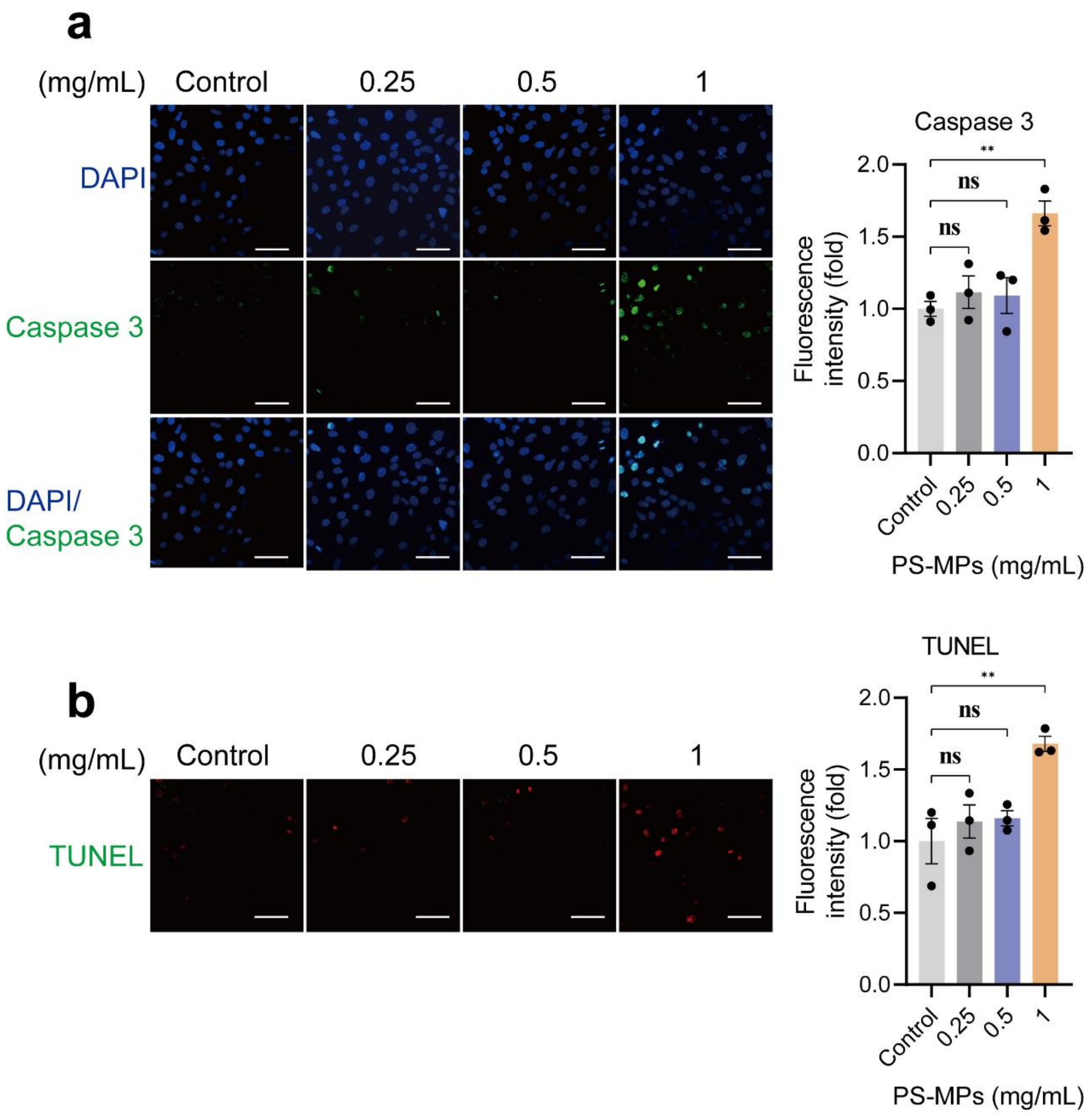

Supplementary Fig.1 Representative IF images showing the expression levels of Caspase3 (a) and TUNEL (b).
