# Supplementary material for: Long-term exposure to polystyrene microplastics triggers premature testicular aging
Source: Part Fibre Toxicol. 2023 Aug 28;20:35. doi: 10.1186/s12989-023-00546-6 (PMC10463354; doi:10.1186/s12989-023-00546-6)
Supplement: Supplementary file 3 — Supplementary Material 3 [file 12989_2023_546_MOESM3_ESM.pdf]

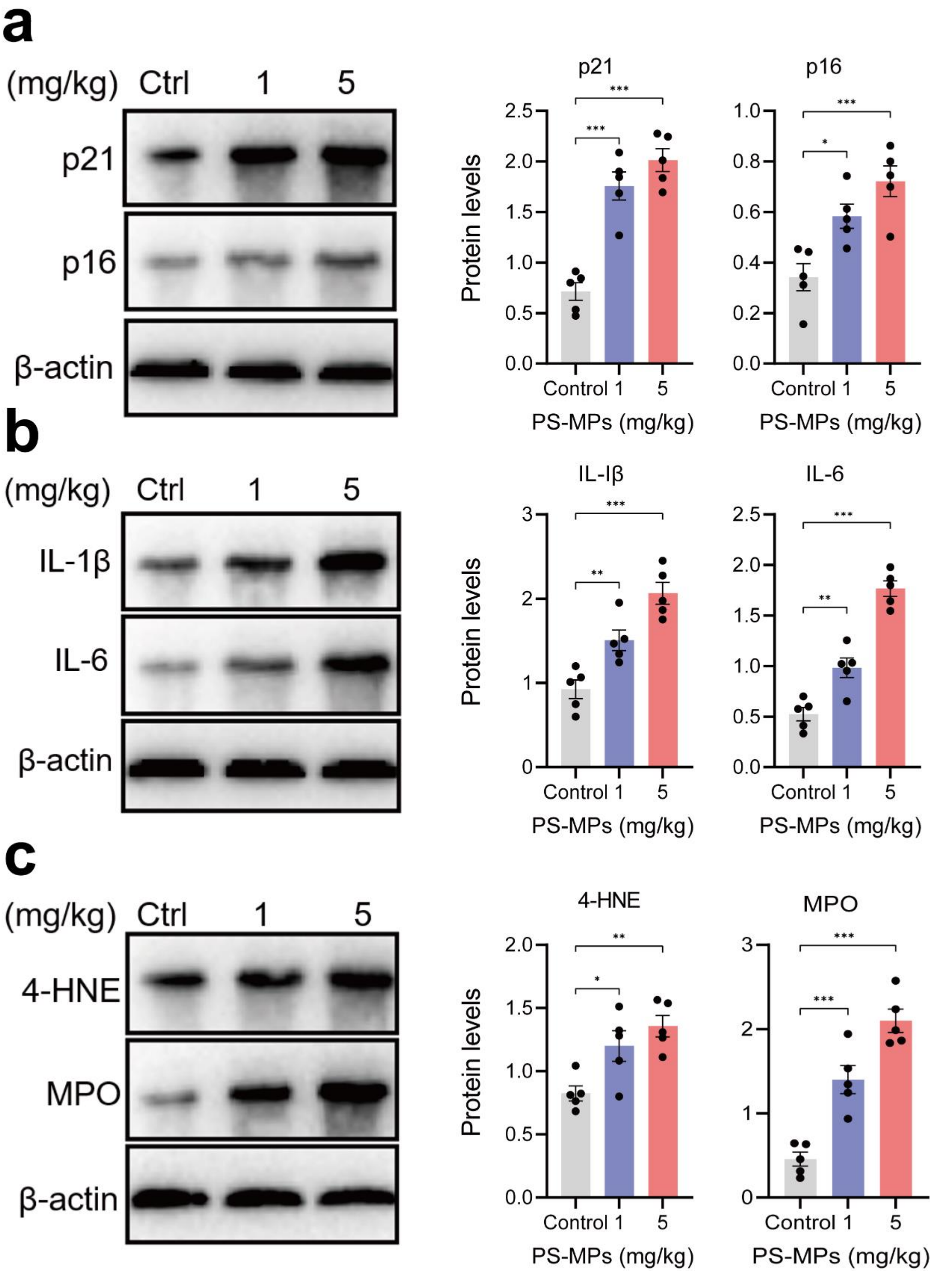

Supplementary Fig.1 Western blot of aging- (**a**), oxidative stress- (**b**)and inflammation- (**c**) related markers.
